# Quadriparesis after CD19 × CD22 bicistronic chimeric antigen receptor T cells for B‐cell acute lymphoblastic leukaemia

**DOI:** 10.1111/bjh.70073

**Published:** 2025-08-25

**Authors:** Alexander W. Rankin, Sara K. Silbert, Brynn B. Duncan, Bonnie Yates, Avindra Nath, Nadia Biassou, Anthony F. Suffredini, Robert L. Danner, Charles Natanson, Amarpreet K. Ahluwalia, Lauren Little, Toni Foley, Hemant S. Murthy, Haneen Shalabi, Paul Nyquist, Nirali N. Shah

**Affiliations:** ^1^ Pediatric Oncology Branch, Center for Cancer Research National Cancer Institute Bethesda Maryland USA; ^2^ National Institute of Neurological Disorders and Stroke National Institutes of Health Bethesda Maryland USA; ^3^ Department of Radiology and Imaging Sciences, National Institutes of Health Clinical Center National Institutes of Health Bethesda Maryland USA; ^4^ Critical Care Medicine Department, National Institutes of Health Clinical Center National Institutes of Health Bethesda Maryland USA; ^5^ National Heart, Lung, and Blood, Institute National Institutes of Health Bethesda Maryland USA; ^6^ Division of Hematology Mayo Clinic Jacksonville Florida USA; ^7^ Department of Neurology Johns Hopkins University School of Medicine Baltimore Maryland USA; ^8^ Neurocritical Care Division, Department of Anesthesiology and Critical Care Medicine Johns Hopkins University School of Medicine Baltimore Maryland USA; ^9^ Present address: Nationwide Children's Hospital Columbus Ohio USA; ^10^ Present address: Novartis Cambridge MA USA

**Keywords:** B‐acute lymphoblastic leukaemia, CAR T cells, neurotoxicity, quadriparesis

To the Editor,

Immune effector cell‐associated neurotoxicity syndrome (ICANS) is seen in 21%–63% of patients receiving CD19 chimeric antigen receptor (CAR) T cells.^1^, ^2^, ^3^ The classic presentation is thought to involve immune insult to the central nervous system (CNS) resulting in symptoms such as aphasia, altered mental status, cognitive impairment, motor weakness, seizures and cerebral oedema.^4^ Risk factors for ICANS include CAR T‐cell co‐stimulatory domain, older age, high disease burden and severe cytokine release syndrome (CRS).^5^, ^6^ CD19 expression on CNS mural cells, targeting of which may facilitate disruption of the blood–brain barrier, has been proposed to be mechanistically linked with the development of ICANS,^7^ highlighting potential on‐target/off‐tumour effects. Here, we describe a patient with relapsed/refractory (r/r) B‐cell acute lymphoblastic leukaemia (B‐ALL) who developed quadriparesis, an emerging variant of ICANS, as a complication of CD19 × CD22 CAR T cells.^8^ Additionally, we provide novel insights into the utility of dasatinib for CAR T‐cell toxicity management.

A 26‐year‐old male with *CRLF2r* chemotherapy‐refractory B‐ALL was referred for CAR T‐cell trial therapy. B‐ALL history included CNS disease at diagnosis. Treatment complications included intrathecal (IT) methotrexate‐related muscle spasms. Due to disease progression and lack of access to commercial CAR T cells, he was referred to the National Cancer Institute (NCI) and enrolled onto a phase I clinical trial of CD19 × CD22 bicistronic CAR T cells (NCT05442515). This construct built upon our prior experiences and was comprised of a CD19 (FMC63) single‐chain variable fragment (scFv) with a CD28 co‐stimulatory domain joined with a CD22 (m971) scFv with a 41BB co‐stimulatory domain.^9^

Bridging therapy with cytarabine and cyclophosphamide was ineffective at disease reduction. Pre‐infusion disease burden showed 93% bone marrow blasts. Cerebrospinal fluid (CSF) was negative by cytology, and despite suspicious rare cells, there was no definitive evidence of B‐ALL within the limit of detection by flow cytometry. There was no extramedullary disease based on brain magnetic resonance imaging (MRI) and ^18^fludeoxyglucose positron emission tomography/computed tomography (PET/CT). Lymphodepletion with fludarabine (75 mg/m^2^) and cyclophosphamide (900 mg/m^2^) was given on days −4 through −2, followed by infusion of autologous CD19 × CD22 CAR T cells at the starting dose of 1 × 10^6^ transduced CAR T cells/kg on day 0.

Fever, heralding CRS onset (American Society of Transplantation and Cellular Therapy [ASTCT] consensus criteria),^4^ developed within hours post‐infusion. Despite a transient response to a single dose of tocilizumab 8 mg/kg on day +1, CRS progressed to grade 3, requiring additional interventions (Figure 1A) including intensive care unit (ICU) management for vasopressor support. Corticosteroids (methylprednisolone 50 mg every 8 h (1.5 mg/kg/day)) were initiated on day +3 and escalated to 100 mg every 8 h (3 mg/kg/day) on day +5. An additional dose of tocilizumab was administered on day +6. Later that day, he developed rapidly progressive severe encephalopathy (immune effector cell‐associated encephalopathy [ICE] score = 1) consistent with ICANS requiring intubation. Methylprednisolone was replaced with dexamethasone, and both emapalumab (1 mg/kg ×1) and high‐dose methylprednisolone (500 mg ×1) were administered. On day +7, despite prophylaxis with levetiracetam, focal seizures developed and were treated with lorazepam and higher dose levetiracetam. Brain MRI on day +7 demonstrated diffuse severe cerebral oedema including the brainstem (Figure 1B,C), at which point siltuximab 11 mg/kg ×1, anakinra and thiamine for encephalopathy^10^ were all initiated alongside higher dosing of dexamethasone (maximum 50 mg every 6 h). With these interventions, by day +9, his mental status was improving, and CRS had fully resolved.

**FIGURE 1:**
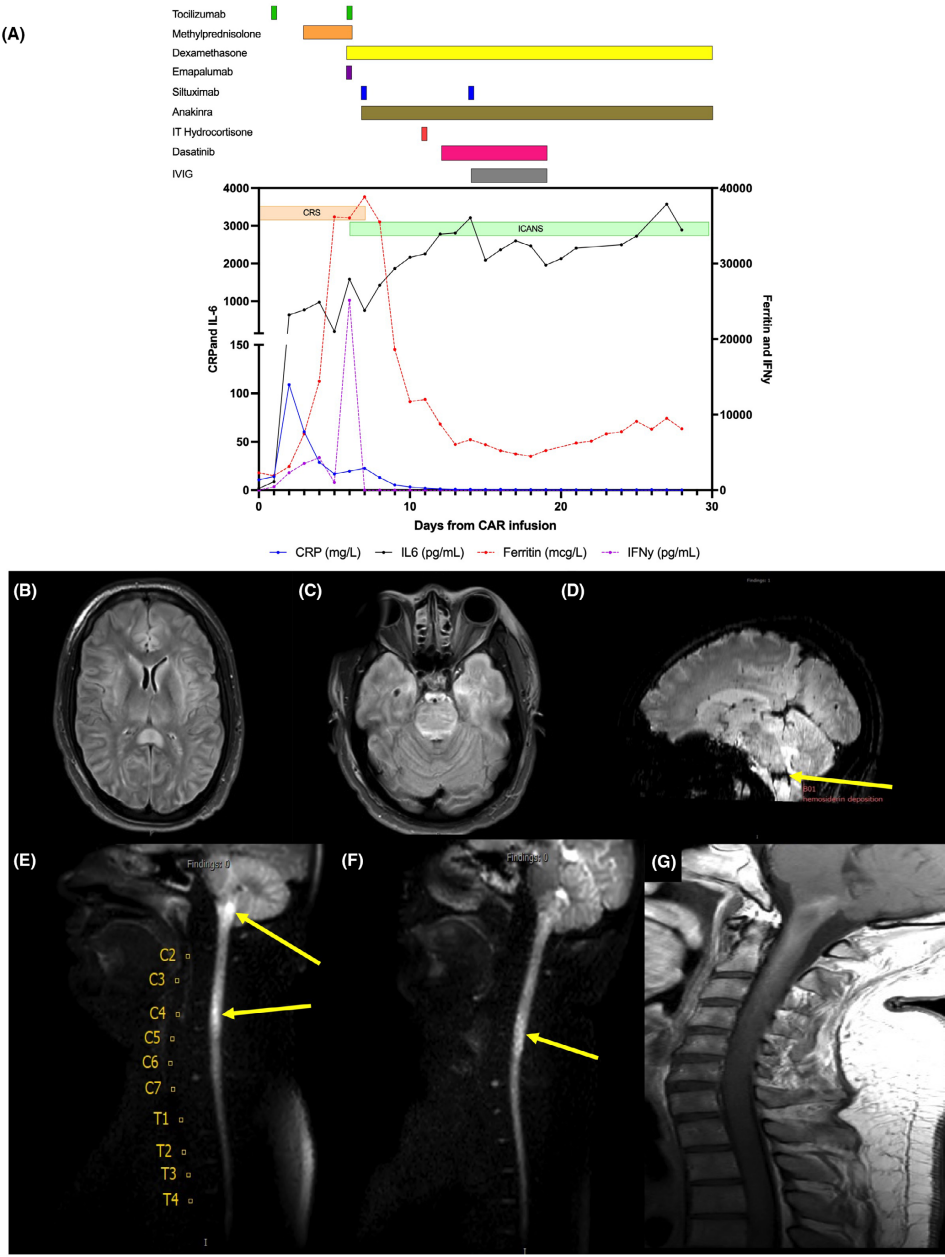
Clinical course and central nervous system imaging. (A) Timeline displaying toxicity course as days from CAR T‐cell infusion on the *x*‐axis (day 0 = infusion). Onset and duration of CRS and ICANS are shown by the shaded bars. Graphical quantification of inflammatory markers including plasma C‐reactive protein (CRP), interleukin (IL)‐6, ferritin and interferon (IFN)‐γ is also shown. Above the graph, pharmacological interventions targeted for toxicity management are displayed; bars represent the timing of respective interventions. (B, C) MRI brain on day +7 showing diffuse cerebral oedema throughout the (B) basal ganglia, insular cortex and cerebrum as well as the (C) pons. (D, E) MRI brain and spine on day +10 showing (D) signal abnormality and haemorrhage in the cervico‐medullary junction suggesting spinal cord ischaemia or infarction as well as (E) diffusion restriction of the spinal cord at the cervico‐medullary junction and at the C4–C5 level. (F, G) MRI spine on day +21 showing (F) persistent restricted diffusion in the cervical cord as well as (G) sagittal T1 precontrast demonstration of the presence of methaemoglobin throughout the spinal cord, suggesting subacute spinal cord haemorrhage. CAR, chimeric antigen receptor; CRS, cytokine release syndrome; ICANS, immune effector cell‐associated neurotoxicity syndrome; MRI, magnetic resonance imaging.

Despite this improvement in mental status and anticipated continued recovery, he was unable to move his extremities. He was unable to be extubated due to ongoing flaccid quadriparesis and concerns for his inability to maintain an open and clear airway. On day +10, brain MRI demonstrated stable to decreasing cerebral oedema, but concurrent MRI of the spinal column revealed a longitudinally extensive lesion involving the cervical cord. The lesion was most prominent at C3 to C5 levels but was contiguous with the brainstem lesion. The lesion involved the entire thickness of the cord. There was diffusion restriction of the cord suggestive of necrosis or ischaemia/infarction (Figure 1D,E) concerning for longitudinal myelitis. Lumbar puncture with IT hydrocortisone for presumptive spinal cord ICANS was performed and a course of dasatinib 100 mg was trialled based on preclinical data suggesting that it may suppress CAR T cells and help mitigate toxicity.^11^ Infectious testing from the blood and CSF was negative. Despite these interventions, alongside ongoing administration of steroids, a month of anakinra, two courses of intravenous immunoglobulin (IVIG) and additional doses of both siltuximab and IT hydrocortisone, spinal cord pathology with restricted diffusion persisted even with cerebral oedema resolving (Figure 1F,G).

Thus, while his mental status returned to baseline, flaccid quadriparesis and neurogenic respiratory failure requiring mechanical ventilation persisted. Restaging evaluations performed on day +25 demonstrated achievement of an minimal residual disease (MRD)‐negative remission with 69% of T cells being CAR‐positive in the bone marrow. Peripheral blood expansion peaked at 82% (of T cells that are CAR+) on day +13 (Figure 2A). Notably, while the initiation of dasatinib decreased the absolute number of circulating CAR T cells, the gradual withdrawal of dasatinib led to a paradoxical increase in absolute expansion (Figure 2B). CSF analysis demonstrated 81% of T cells as CAR positive on day +11, with ongoing CAR T‐cell presence up through final sampling on day +48 at 69%. While CAR T cells were no longer detectable by flow cytometry in the blood or bone marrow by 3 months after infusion, the patient remains in an ongoing MRD‐negative complete remission nearly 2 years post‐infusion, albeit with ongoing quadriparesis, ventilator dependence and transition to hospice to focus on comfort and quality of life.

**FIGURE 2:**
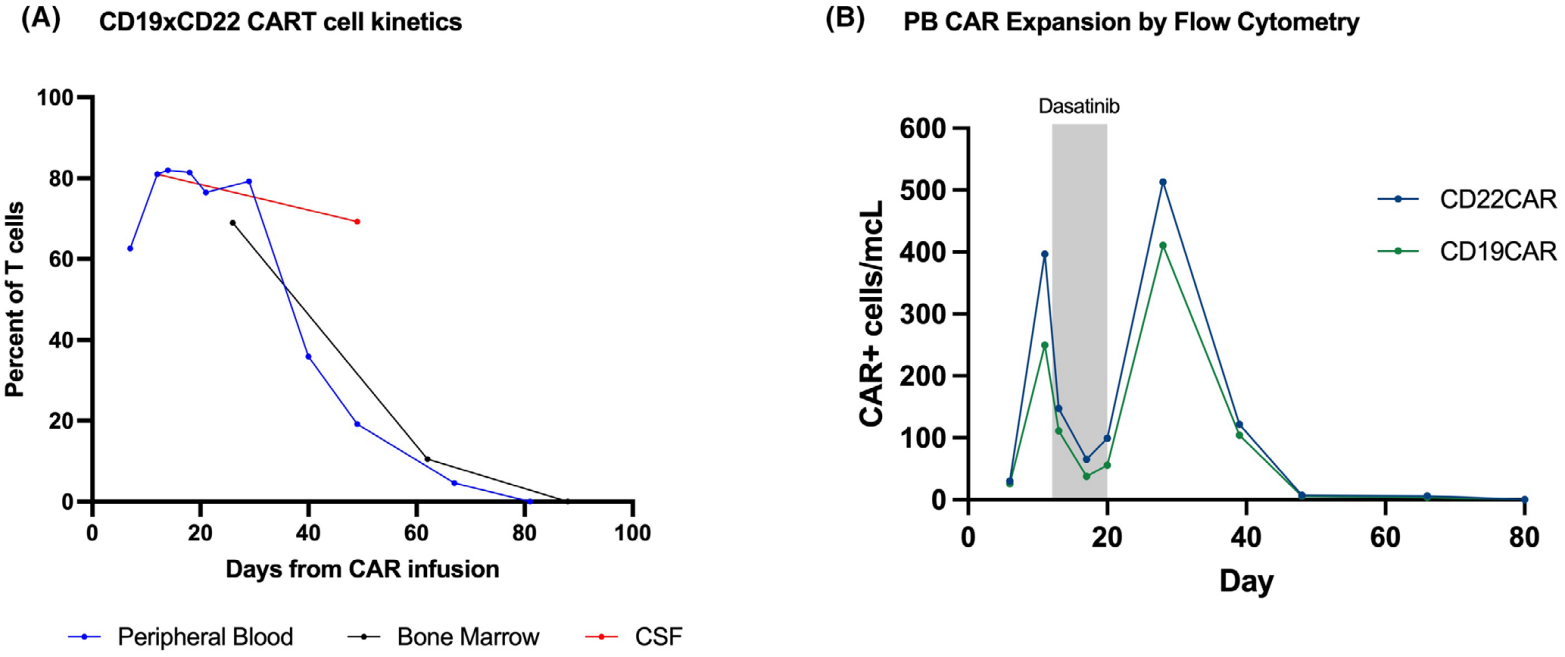
CAR T‐cell expansion & peripheral blood CAR expansion by flow cytometry. (A) Graphical quantification of CAR T cells as a percentage of total T cells present in the peripheral blood, bone marrow and CSF. The *x*‐axis represents days from CAR T‐cell infusion (day 0 = infusion). CSF white blood cell count was 1/mm^3^ at both time points. (B) Dasatinib was started on day +12 and then tapered off from day +18 through +20 in the hope of slowing CAR expansion and associated toxicities. On day +11, CD22 CAR T cells were detected at 396.5 cells/mL in the peripheral blood, decreasing to a nadir of 65 cells/mL on day +17. As dasatinib doses decreased, CAR T cells increased to 99.4 cells/mL on day +20 followed by a peak of 513.3 CD22 CAR T cells/mL on day +28. CAR, chimeric antigen receptor; CSF, cerebrospinal fluid.

As experience with adoptive cell therapies grows, novel forms of neurotoxicity with presentations that diverge from classical ICANs are emerging. Multiple groups have now reported cases of post‐CAR T‐cell myelopathy and paresis that often result in prolonged neurological deficits and appear to occasionally be irreversible.^8^, ^12^ Deschenes‐Simard et al. recently reviewed 18 cases reported in the literature, with 44.4% having no and 33.3% having partial clinical improvement.^8^ Many reported cases have occurred in adults receiving CAR T cells for B‐cell lymphoma^8^; however, several cases reported recently by Diorio et al. are in younger patients with B‐ALL as well.^12^ These included two patients who had received CD22‐targeted CAR T cells, suggesting this is not limited to CD19 targeting. To our knowledge, our patient is the first reported to develop myelopathy and quadriparesis after dual CD19 and CD22 targeting.

Many cases of post‐CAR myelopathy appear to be preceded by or occur concurrently with severe CRS and ICANs.^12^ Additionally, the diagnosis of paresis may be obscured or delayed due to ongoing encephalopathy, as in our case. Robust CAR T‐cell expansion and hyperinflammation (particularly with CD28 co‐stimulatory containing CAR T‐cells) are associated with severe neurotoxicity^1^ and may promote spinal cord inflammation. However, the prolonged spinal cord damage, even with the resolution of other ICANs symptoms, points to other pathophysiological mechanisms such as spinal cord infarction,^13^ direct anti‐tumour effect in those with CNS involvement, on‐target/off‐tumour toxicity^7^, ^14^ or infection.^12^ (Table 1).

**TABLE 1:** Differential diagnosis of quadriparesis after CAR T cells.

CNS infection
Critical illness polyneuropathy/myopathy
Demyelinating disorders (MS, ADEM)
Eosinophilic myelitis
Guillain–Barre syndrome
On‐target off‐tumour toxicity
Transverse myelitis (infectious, immune‐mediated)
Tumour inflammation‐associated neurotoxicity
Severe presentation of ICANS
Stroke (ischaemic or haemorrhagic)
Toxicity of intrathecal chemotherapy

Abbreviations: ADEM, acute demyelinating encephalomyelitis; CAR, chimeric antigen receptor; CNS, central nervous system; ICANS, immune effector cell‐associated neurotoxicity syndrome; MS, multiple sclerosis.

While our patient did have significant CAR T‐cell expansion with severe CRS and ICANs, there was no apparent risk factor predisposing to myelopathy and quadriparesis, such as prior exposure to CNS radiation^15^ or an identifiable infectious trigger.^16^, ^17^ Similar to recent cases, we hypothesize that the same inflammatory process that led to cerebral oedema (and was fully responsive to ICANS directed interventions) also developed in the spinal cord. This resulted in cord oedema and swelling that, in the confined and restrictive enclosure of the spinal canal, resulted in infarction and irreversible damage not responsive to interventions.

One notable observation in this case relates to the biphasic CAR T‐cell expansion. While multiple potential explanations may exist (including incorporation of two unique single‐chain variable fragments (scFv) and co‐stimulatory domains), dasatinib exposure could lead to transient inhibition of CAR signalling^18^ thus hypothetically enabling a more robust secondary expansion upon removal of the drug. The timing of the decreased CAR T‐cell detection in peripheral blood and subsequent re‐expansion aligns with dasatinib dosing and cessation respectively. This warrants further study of its use in toxicity mitigation, especially as there was no apparent benefit and biphasic CAR T‐cell expansion has not been seen in other patients on this trial.

In our experience and that of others, CD22 CAR T cells are associated with a lower risk of neurotoxicity.^12^, ^19^, ^20^, ^21^ Whether dual‐targeting augments toxicity remains under study. Additionally, incorporation of both CD28 and 4‐1BB costimulatory domains in this bicistronic construct raises intriguing questions related to CAR signalling and activation that warrant further research. Given the association of neurotoxicity with high disease burden, we amended our trial to dose reduce to dose level −1 (3 × 10^5^ transduced CAR T cells/kg) and established high‐ and low‐disease burden treatment cohorts. To date, no further patients (*n* = 5), including one with active CNS2 disease, have developed neurotoxicity, myelopathy or quadriparesis.

In conclusion, CAR T‐cell‐associated quadriparesis is a rare but consequential complication that requires a high clinical index of suspicion and close monitoring. Why this process seems to involve the spinal cord in some patients and not others requires further study. Importantly, spinal imaging should be pursued in those who develop sensory or motor deficits, and consideration of alternate aetiologies (i.e. infection) as part of the diagnostic workup is prudent. As access to CAR T cells expands to an ever larger cohort of patients and the library of available constructs grows, understanding novel toxicities will be crucial to ensuring that this revolutionary therapy remains safe and effective.

## AUTHOR CONTRIBUTIONS

A.W.R. and N.N.S. wrote the first version of this manuscript. A.W.R., S.K.S., B.B.D., B.Y. and N.N.S. provided critical input on the manuscript draft, including on the analysis of the biological correlatives and treatment course. S.K.S., B.Y., A.N., A.F.S., R.L.D., C.N., A.K.A., L.L., T.F., H.S.M., H.S., P.N. and N.N.S. all were directly involved with the patient and the care he received, including critical care and neurological evaluation and/or other support based on the area of expertise. N.B. provided insights into radiographic findings. All authors contributed significantly to the manuscript draft and agreed with the submission.

## CONFLICT OF INTEREST STATEMENT

This work was supported in part by the Intramural Research Program of the National Institutes of Health, National Cancer Institute, Center for Cancer Research and the Warren Grant Magnuson Clinical Center (ZIA BC 012187, N. Shah). N.N.S. receives research funding from Lentigen, VOR Bio and CARGO therapeutics and has participated in Advisory Boards for Sobi, Allogene, invoX, ImmunoACT and VOR. H.S.M. is on Advisory Boards/Consultancy for CRISPR Therapeutics, BMS, Jazz, Incyte, Sobi, Autolus and Senti Bioscience and is a medical monitor for BMT CTN.

## DISCLAIMER

This research was supported [in part] by the Intramural Research Program of the National Institutes of Health (NIH). The contributions of the NIH author(s) were made as part of their official duties as NIH federal employees, are in compliance with agency policy requirements and are considered Works of the United States Government. However, the findings and conclusions presented in this paper are those of the author(s) and do not necessarily reflect the views of the NIH or the U.S. Department of Health and Human Services.

## Data Availability

De‐identified participant data that support the findings of this article will be shared upon approved written request to researchers who provide a methodologically sound proposal for the purposes of achieving specific aims outlined in that proposal. To gain access, data requesters will need to sign a data access agreement and to confirm that data will only be used for the agreed purpose for which access was granted. Requests can be directed to Nirali N. Shah, MD: nirali.shah@nih.gov.
